# Extracorporeal Irradiation and Reimplantation with Total Hip Arthroplasty for Periacetabular Pelvic Resections: A Review of 9 Cases

**DOI:** 10.1155/2016/2549616

**Published:** 2016-04-20

**Authors:** Lester Wai Mon Chan, Jungo Imanishi, Samuel Y. Ngan, Sarat Chander, Julie Chu, Renae Thorson, Grant Pang, Peter Choong

**Affiliations:** ^1^Department of Orthopaedics, St. Vincent's Hospital, Melbourne, VIC 3065, Australia; ^2^Department of Orthopaedic Surgery, Tan Tock Seng Hospital, 11 Jalan Tan Tock Seng, Singapore 308433; ^3^Division of Radiation Oncology, Peter MacCallum Cancer Centre, East Melbourne, VIC 3002, Australia; ^4^Bone and Soft Tissue Sarcoma Unit, Peter MacCallum Cancer Centre, East Melbourne, VIC 3002, Australia; ^5^Department of Surgery, University of Melbourne, St. Vincent's Hospital, Fitzroy, VIC 3065, Australia

## Abstract

We report the early results of nine patients with periacetabular malignancies treated with Enneking and Dunham type 2 resection and reconstruction using extracorporeally irradiated (ECI) tumour bone combined with total hip arthroplasty (THA). Diagnosis was chondrosarcoma in six patients, osteosarcoma in two patients, and metastatic renal cell carcinoma in one patient. All patients underwent surgical resection and the resected specimen was irradiated with 50 Gy in a single fraction before being prepared for reimplantation as a composite autograft. The mean follow-up was 21 months (range, 3–59). All patients were alive at latest follow-up. No local recurrence was observed. One patient serially developed three pulmonary metastases, all of which were resected. One experienced hip dislocation due to incorrect seating of an acetabular liner. This was successfully treated with revision of the liner with no further episodes of instability. There were no cases of deep infection or loss of graft. The average Musculoskeletal Tumor Society (MSTS) score was 75% (range, 57–87%). Type 2 pelvic reconstruction with ECI and THA has shown excellent early oncological and functional results in our series. Preservation of the gluteus maximus and hip abductors is important for joint stability and prevention of infection.

## 1. Introduction

Operations for pelvic sarcomas are among the most technically challenging procedures facing orthopaedic oncology surgeons. The priority of surgical management is wide resection with negative margins. Advances in imaging, chemo- and radiotherapy, and surgical technique have allowed limb salvage to become increasingly common. Pelvic reconstruction is difficult and results have often been disappointing [1–6]. Limited function and a high rate of complications including infection, dislocation, prosthetic loosening, and failure of limb salvage are typical. For this reason some authors advocate iliofemoral fusion or resection without formal reconstruction preferring to tolerate limb shortening for a lower risk of complication [7, 8].

Pelvic sarcoma is a heterogenous group in terms of patient characteristics, anatomical extent, and tumour biology. There is no “one-size-fits-all” solution for resection or reconstruction and the indications for limb salvage and the types of reconstruction are variable between centers and surgeons. Pelvic sarcoma surgery is resource intensive with high potential morbidity. For this reason, identifying the group of patients that are most likely to benefit from a particular surgical strategy is of great importance. Extracorporeal irradiation (ECI) and reimplantation of tumour bone is a technique employed by some centers; however, existing series suffer from marked heterogeneity making interpretation of results difficult [9–12].

We have been selectively employing ECI as a reconstructive strategy since 2010. In this series we report our early results in a homogenous group of Enneking and Dunham type 2 pelvic resections [13] treated with the same reconstructive strategy of extracorporeal irradiation and reimplantation with composite total hip arthroplasty (THA).

## 2. Materials and Methods

Ethics approval for this study was obtained from St. Vincent's Hospital.

A list of 23 consecutive patients who underwent ECI was obtained from the database of the Bone and Soft Tissue Sarcoma Service at St. Vincent's Hospital. Eleven patients had ECI of pelvic tumours and 12 had ECI to extremity tumours. Of the 11 patients with pelvic surgery, 9 patients underwent type 2 hemipelvectomy and reconstruction with total hip arthroplasty; one patient underwent a type 1 iliac resection and the other underwent a bilateral type 3 resection of the pubis.

Of the 9 patients with type 2 resections there were 5 male and 4 female patients. The mean age at diagnosis was 51 (range, 40–67). The mean follow-up was 21 months (range, 3–59 months). The histological diagnosis was chondrosarcoma in 6 cases, osteosarcoma in 2 cases, and solitary metastatic renal cell carcinoma in 1 case.

**Figure 1:**
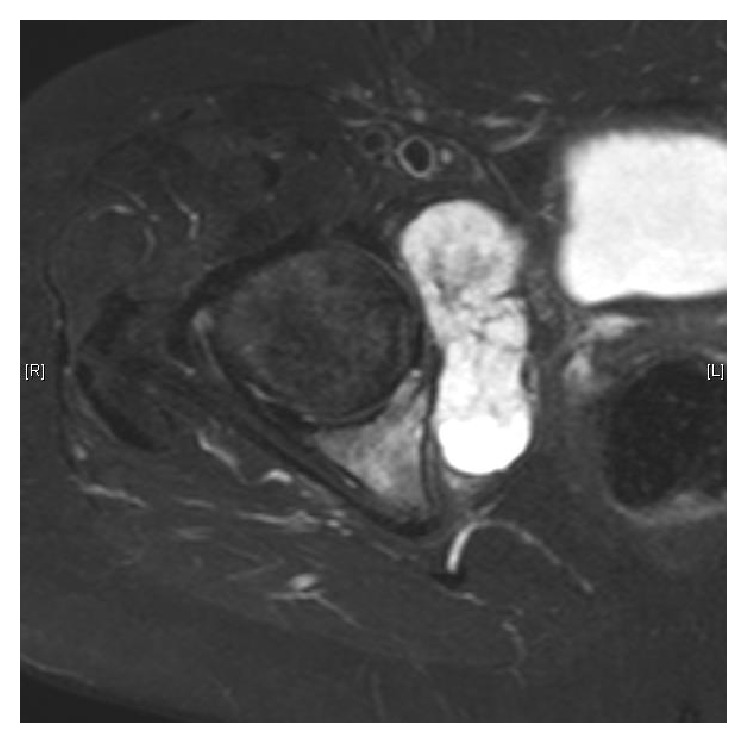
Periacetabular chondrosarcoma with extraosseous extension.

In 4 cases the tumour was completely intraosseous; 5 cases had extraosseous extension (see Figure 1). One case was previously treated with intralesional curettage at another institution. No patients had metastasis at diagnosis.

### 2.1. Procedure

All patients underwent wide en bloc resection of the pelvic tumour. The antibiotic regimen consisted of intravenous 2 g cefazolin and 1 g vancomycin at induction of anaesthesia and was readministered at 6 hours intraoperatively. Antifibrinolytics were not used with the exception of the most recent patient (case 9) who received 1 g tranexamic acid prior to the pelvic osteotomies.

An extensile Y incision (iliopubic and iliofemoral) or modified posteriorly based C (iliofemoral) incision was used. The gluteus maximus was detached from the iliac crest and reflected posteriorly with the overlying skin. Vascularity from the superior and inferior gluteal vessels was preserved. Wide lateral exposure was achieved with a greater trochanter osteotomy with preservation of the majority of the hip abductor origin on the iliac crest. Anterior exposure was achieved with detachment of the inguinal ligament with a sliver of bone from the anterior superior iliac spine (ASIS).

After mobilization of anterior and posterior nerves, vessels, and soft tissues, osteotomies were made at the appropriate levels in the ilium and pubic rami/ischium and the femoral neck. The specimen was removed en bloc with wide tumour margins. Computer navigation was used to determine osteotomy cuts in 2 patients.

With a separate set of instruments, gross tumour and soft tissue was removed from the specimen. This was then wrapped in moist gauze and sealed in a sterile plastic bag, followed by a second layer of moist gauze and sterile plastic. This was wrapped in a third layer of dry cloth and a nonsterile outer plastic bag. Excess air was removed from each of the layers. The specimen was transported to the radiation facility at the Peter MacCallum Cancer Centre (PMCC). Upon arrival, the specimen was packed into a PVC box surrounded by intravenous fluid bags that served as tissue equivalent material. A standard plan was used for all patients and the entire box including the specimen was irradiated to a total dose of 50 Gy in a single fraction. Packing the specimen with tissue equivalent material allows a “build-up” effect of the radiation beam to occur before it hits the bone surface (empty air spaces can potentially attenuate the dose); this allows the delivery of a homogenous dose to the entire specimen [14]. The irradiated bone was transported back to the operating theatre and unpacked under sterile conditions. The total time from the specimen leaving the theatre to return was approximately 1 hour. This was comprised of 20-minute transport in each direction, 5 minutes for packing and unpacking the box, and 10 minutes for delivery of radiation. On return to theatre the specimen was soaked in a solution of alcoholic iodine and prepared for reimplantation with removal of any remaining soft tissue and tumour and filling of defects with cement as necessary. The acetabulum was reamed and prepared for a cemented cup-cage construct. One patient had a dual mobility hip system where the outer acetabular liner was secured to the cage via a screw. The specimen was reimplanted and fixed to the osteotomy sites with screws and plates. The acetabular cage was secured into place and a polyethylene cup was cemented into place. A hip prosthesis was inserted into the proximal femur. Following relocation of the hip joint, the greater trochanter was fixed with a cable grip system and the joint checked for stability. The wound was then thoroughly irrigated with pulsatile lavage. Gluteus maximus and abdominal musculature were reattached to the iliac crest and the inguinal ligament reattached to the ASIS. Closure was completed over drains. Mean operative duration from incision to wound closure was 7:27 (range, 5:10–11:30).

Postoperatively, drains were removed at days 3–7 depending on drain output. Intravenous cefazolin (2 g/8 hr) and vancomycin (dose adjusted according to trough levels) were continued until drains were removed and subsequently converted to oral cephalexin (500 mg/6 hr), which was given until 2 weeks after surgery. Prophylactic doses of subcutaneous low molecular weight heparin were started on postoperative day 1 unless there were concerns regarding bleeding or postoperative coagulopathy. The patient was fitted with an abduction brace and instructed to be non-weight-bearing for 3 months after which progressive weight bearing with physiotherapy supervision was permitted.

## 3. Results

A summary of our cases is shown in Table 1.

### 3.1. Local Recurrence

There were no local recurrences in our series.

### 3.2. Distant Metastasis

One patient with osteosarcoma developed a single pulmonary metastasis at 12 months after surgery, which was treated with pulmonary metastasectomy. He subsequently developed further pulmonary metastases that were resected at 21 and 30 months after initial surgery. He is currently alive with no evidence of disease at 36 months.

### 3.3. Death

There were no perioperative deaths.

### 3.4. Union

**Figure 2:**
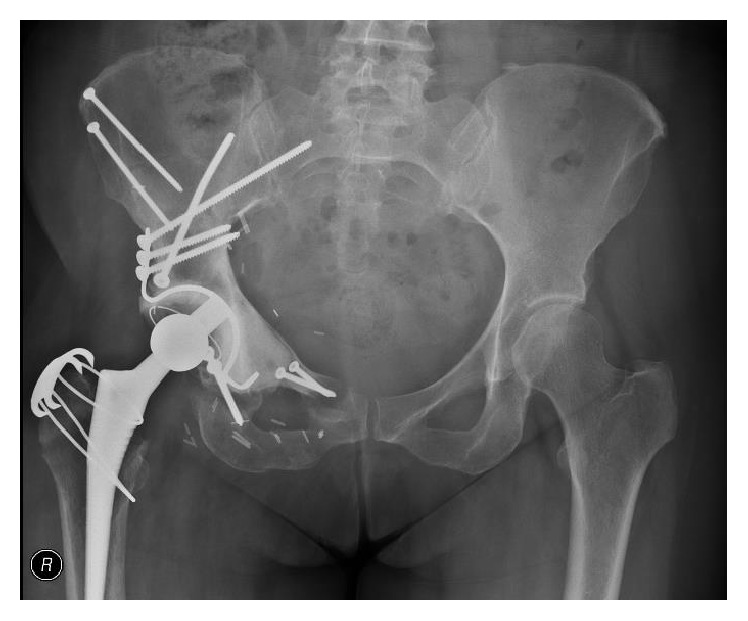
Postoperative X-ray at 6 months. Asymptomatic nonunion is seen at the ischial and pubic osteotomies. The configuration of screws and plates and screws through flanged acetabular cages was variable between cases.

Assessment of union in pelvic resections is technically difficult. X-rays are unreliable and CT scans suffer from metal artifact and were not routinely done. All patients with more than 6-month follow-up appeared to have united at the iliac osteotomy site. Two patients appeared to have radiographic nonunion at the pubic and/or ischial osteotomy; however no intervention has been required for this (see Figure 2).

### 3.5. Hip Dislocation

One patient had recurrent dislocation of his total hip arthroplasty due to an incorrectly seated acetabular liner from a dual mobility hip. Revision surgery was undertaken at 5 months after the index procedure. At surgery it was apparent that the liner had not locked because of cement in the locking screw threads. Cement was removed and a new liner was inserted. After revision the patient had no further episodes of instability but did represent at 2 weeks with a symptomatic pulmonary embolus and deep vein thrombosis of the operated leg. This was treated with anticoagulation and the patient has not suffered any long-term sequelae.

### 3.6. Infection and Wound Breakdown

There were no episodes of infection or other wound complications. To date all patients have retained their grafts and there have been no episodes of implant loosening or failure.

### 3.7. Functional Outcome

All patients with more than 1-year follow-up were assessed with a Musculoskeletal Tumor Society (MSTS) score. Seven patients were assessed. The mean MSTS score was 75 (range, 57–87). Six of 7 were able to walk independently without the use of walking aids. One patient has returned to horse riding and one patient had a successful pregnancy with delivery by elective caesarian section at 38 weeks.

## 4. Discussion

Periacetabular reconstruction is challenging and no single reconstructive strategy has been shown to be superior. Anatomical reconstruction of a functional hip joint seems desirable but attempts have often resulted in a high complication rate. The saddle prosthesis was initially popular due to ease of insertion and proposed benefits of preservation of length and hip mobility compared with fusion. It has since fallen out of favour with reports of poor function and high complication rates [1]. Later generations of endoprosthesis that relied upon fixation to the remnant ilium have also suffered from high rates of instability, loosening, and infection [2–5].

Nonanatomical methods of reconstruction accept a shortened limb and abnormal biomechanics. Successful iliofemoral fusion results in reasonable function but may be associated with later dysfunction of the lumbar spine and knee. Failure of fusion and development of a pseudarthrosis is associated with poorer outcomes [7]. Hip transposition with endoprosthesis and pseudo-capsule reconstruction with a reconstructive mesh tube has been reported with modest functional results with a MSTS score of 62% and deep infection in 32% of patients [15]. Alternatively, resection without bone reconstruction has been reported with surprising good functional results and low complication rates. Average MSTS scores in one series were 73% [8].

Biological reconstruction with bulk allograft is well described but requires access to a large bone bank [16]. Even at centers with such a facility there may be difficulty obtaining a suitably matched donor bone and immunogenicity and transmission of infection are concerns. Recycled autograft is an appealing option as it provides the inherent benefits of a perfect fit, low cost reconstruction while obviating concerns regarding disease transmission. With successful osteointegration, autograft may result in a highly durable reconstruction. The possibility of reimplanting viable tumour cells is a concern; however, there are no reports of local recurrence occurring within the irradiated bone at doses between 50 and 300 Gy [9–12].

Recycled irradiated autograft was first reported by Spira and Lubin in 1968 [17] and has become a well-recognized method of reconstruction. There are limited series specifically reporting ECI in pelvic reconstruction and even less information specifically regarding periacetabular reconstruction.

Hong et al. report the largest series of 35 pelvic reconstructions using ECI as part of their total series of 101 cases. They did not report the type of resection or the details of their reconstruction [12].

Wafa et al. reported 18 cases of pelvic ECI. The majority of their reconstructions were for type I-II resections in a younger patient population with a high proportion of osteosarcoma and Ewing's sarcoma. Nine patients (50%) died from metastatic disease, 3 patients (17%) had local recurrence, and 3 patients had deep infection that required removal of graft in 2 cases and hindquarter amputation in 1. Function was reasonable with a mean MSTS score of 77% [9].

Krieg et al. reported 13 cases of pelvic Ewing's sarcoma treated with ECI with excellent function outcome. Again this was in a young patient population. Four patients died of disease. Of note, 6 patients aged 13–29 were treated with a composite periacetabular autograft with total hip arthroplasty. Three patients (23%) had complications requiring further surgery including one wound necrosis, one patient who had a fractured autograft treated conservatively, and subsequent acetabular cup loosening requiring revision, and one deep infection requiring removal of graft [18].

Sys et al. reported 15 cases treated with pelvic ECI at a dose of 300 Gy. 13/15 patients experienced complications. This included 7 patients who died following local recurrence and 3 infections (20%). Five patients had a composite total hip arthroplasty of whom 2 patients experienced recurrent dislocations. One patient had nonunion and loss of fixation [10].

In contrast to the existing literature, our indications for ECI reconstruction appear to be more limited. All patients in this series had tumours that were amenable to a type 2 resection with preservation of the iliac wing. We believe that the importance of this is twofold. (1) The iliac osteotomy from below the anterior superior iliac spine to the greater sciatic notch provides a good surface for reintegration and is very amenable to stable osteosynthesis with plates and screws. (2) Repair of the gluteus maximus onto the iliac crest and preservation of the majority of the hip abductors provides stability for the hip joint and protection from infection via the physical barrier of a robust muscular envelope, improved vascularity to the area surrounding the irradiated bone, and minimization of dead space. No patient in this series experienced a deep infection and we attribute this to the factors described above as well as strict adherence to perioperative protocols including the extended use of intravenous and oral antibiotics and careful aseptic handling of the irradiated bone to avoid contamination and the soaking of the bone in alcoholic iodine on return to theatre.

All patients had a total hip replacement as part of their reconstruction and one patient had dislocation. Ironically, this was the only patient in which a dual mobility hip implant was used for the theoretical advantage of improved stability. We consider the incorrect fixation of the liner in this case to be a technical error rather than a problem inherent to the reconstructive strategy.

It should be noted that our case mix was comprised of a majority of patients with chondrosarcoma. Only 2 patients with osteosarcoma and 1 patient with renal cell carcinoma were treated with systemic chemotherapy. This may also have contributed to a low rate of infection and wound complications in comparison to other studies. This also accounts for the high survival rate and low rate of distant metastases as chondrosarcoma has a lower tendency to metastasize than other bone sarcomas.

The dose of radiotherapy required for adequate sterilization of tumour bone is controversial. There are no reports of recurrence within ECI treated bone although local recurrence in the surrounding soft tissue is reported. We had no local recurrences in our series to date, supporting our current treatment strategy of using 50 Gy. Higher doses are associated with detrimental effects on the structure and biology of the irradiated bone [19–21]. As described in our procedure section, the creation of a surrounding interface of tissue equivalent material improves delivery of radiation and special attention should be paid to the preparation of the bone [14]. The materials, protocols, and procedures required for this must be prepared in advance of surgery.

**Figure 3:**
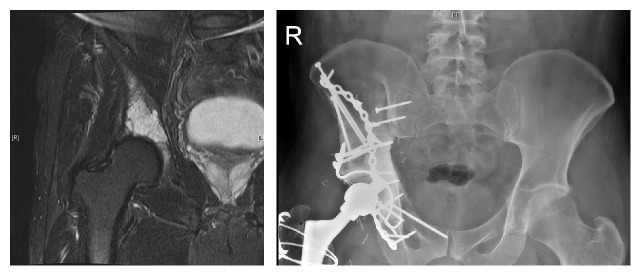
Patient number 4 had poorer functional outcome (MSTS 57). This may be accounted for by the higher level iliac resection and more extensive stripping of the abductor musculature.

Function in our series was generally excellent. For patients with more than 1-year follow-up, 6 of 7 patients were independently walking without the use of a crutch or brace. All patients had a noticeable limp. One patient (case 4) had a lower MSTS score of 57. In this case, tumour extended higher into the ilium and required more extensive stripping of the abductor musculature and a higher osteotomy than in the other cases (see Figure 3). We postulate that this may account for the poorer functional score.

## 5. Conclusion

Extracorporeal irradiation and reimplantation of autograft with total hip arthroplasty is an excellent option for reconstruction of type 2 defects following resection of periacetabular sarcomas. Functional results are very good with a low rate of complication. Preservation of the gluteal musculature is important for soft tissue coverage and prevention of infection and joint stability and function.

## Figures and Tables

**Table 1:** Summary of cases.

Number	Gender	Age	Histo./Grade	Compartment	Follow-up (mths)	Complications	Recurrence/metastasis	MSTS score	Status
1	Male	49	OS/1	Extracompartmental^*∗*^	59	No	None	70	CDF
2	Male	44	OS/3	Intracompartmental	35	No	Lung metastasis—metastasectomies done	87	NED
3	Male	40	CS/2	Extracompartmental	19	Dislocation, acetabular liner revised, DVT/PE, and heterotopic ossification	None	83	CDF
4	Male	56	CS/2	Intracompartmental	16	No	None	57	CDF
5	Female	59	RCC	Extracompartmental	18	No	None	70	CDF
6	Female	44	CS/1	Intracompartmental	17	No	None	77	CDF
7	Female	55	CS/2	Extracompartmental	14	No	None	83	CDF
8	Male	50	CS/2	Intracompartmental	6	No	None	N/A	CDF
9	Female	67	CS/2-3	Extracompartmental	3	No	None	N/A	CDF

OS: osteosarcoma, CS: chondrosarcoma, RCC: renal cell carcinoma, DVT/PE: deep vein thrombosis/pulmonary embolism, CDF: Continuously Disease-Free, and NED: no evidence of disease. ^*∗*^Low grade central osteosarcoma—extraosseous extension was due to prior intralesional curettage.
